# Enhancing Suicide Risk Prediction With Polygenic Scores in Psychiatric Emergency Settings: Prospective Study

**DOI:** 10.2196/58357

**Published:** 2024-10-23

**Authors:** Younga Heather Lee, Yingzhe Zhang, Chris J Kennedy, Travis T Mallard, Zhaowen Liu, Phuong Linh Vu, Yen-Chen Anne Feng, Tian Ge, Maria V Petukhova, Ronald C Kessler, Matthew K Nock, Jordan W Smoller

**Affiliations:** 1 Psychiatric & Neurodevelopmental Genetics Unit Center for Genomic Medicine Massachusetts General Hospital Boston, MA United States; 2 Stanley Center for Psychiatric Research Broad Institute of MIT and Harvard Cambridge, MA United States; 3 Department of Psychiatry Harvard Medical School Boston, MA United States; 4 Department of Epidemiology Harvard T. H. Chan School of Public Health Boston, MA United States; 5 Center for Precision Psychiatry Massachusetts General Hospital Boston, MA United States; 6 School of Computer Science Northwestern Polytechnical University Xi’an China; 7 Harvard College Harvard University Cambridge, MA United States; 8 Institute of Health Data Analytics and Statistics College of Public Health National Taiwan University Taipei Taiwan; 9 Department of Health Care Policy Harvard Medical School Boston, MA United States; 10 Department of Psychology Harvard University Cambridge, MA United States; 11 Mental Health Research Program Franciscan Children’s Brighton, MA United States; 12 Department of Psychiatry Massachusetts General Hospital Boston, MA United States

**Keywords:** polygenic risk score, suicide risk prediction, suicide attempt, predictive algorithms, genomics, genotypes, electronic health record, machine learning

## Abstract

**Background:**

Despite growing interest in the clinical translation of polygenic risk scores (PRSs), it remains uncertain to what extent genomic information can enhance the prediction of psychiatric outcomes beyond the data collected during clinical visits alone.

**Objective:**

This study aimed to assess the clinical utility of incorporating PRSs into a suicide risk prediction model trained on electronic health records (EHRs) and patient-reported surveys among patients admitted to the emergency department.

**Methods:**

Study participants were recruited from the psychiatric emergency department at Massachusetts General Hospital. There were 333 adult patients of European ancestry who had high-quality genotype data available through their participation in the Mass General Brigham Biobank. Multiple neuropsychiatric PRSs were added to a previously validated suicide prediction model in a prospective cohort enrolled between February 4, 2015, and March 13, 2017. Data analysis was performed from July 11, 2022, to August 31, 2023. Suicide attempt was defined using diagnostic codes from longitudinal EHRs combined with 6-month follow-up surveys. The clinical risk score for suicide attempt was calculated from an ensemble model trained using an EHR-based suicide risk score and a brief survey, and it was subsequently used to define the baseline model. We generated PRSs for depression, bipolar disorder, schizophrenia, suicide attempt, and externalizing traits using a Bayesian polygenic scoring method for European ancestry participants. Model performance was evaluated using area under the receiver operator curve (AUC), area under the precision-recall curve, and positive predictive values.

**Results:**

Of the 333 patients (n=178, 53.5% male; mean age 36.8, SD 13.6 years; n=333, 100% non-Hispanic and n=324, 97.3% self-reported White), 28 (8.4%) had a suicide attempt within 6 months. Adding either the schizophrenia PRS or all PRSs to the baseline model resulted in the numerically highest discrimination (AUC 0.86, 95% CI 0.73-0.99) compared to the baseline model (AUC 0.84, 95% Cl 0.70-0.98). However, the improvement in model performance was not statistically significant.

**Conclusions:**

In this study, incorporating genomic information into clinical prediction models for suicide attempt did not improve patient risk stratification. Larger studies that include more diverse participants are required to validate whether the inclusion of psychiatric PRSs in clinical prediction models can enhance the stratification of patients at risk of suicide attempts.

## Introduction

Between 2000 and 2018, suicide rates increased by 37%, making suicide one of the leading causes of death in the United States [1]. Data from US health care systems show that most individuals who die by suicide in the United States had health care visits in the month preceding their death, highlighting opportunities for health care providers to identify and intervene with those at risk for suicide-related behavior [2].

We previously developed and validated a prognostic model combining electronic health records (EHRs) and a brief patient-reported survey that was able to prospectively predict short-term risk for suicide attempts after an emergency department (ED) visit for psychiatric problems [3]. This study was designed to extend our previous work by evaluating whether adding polygenic risk scores (PRSs) for neuropsychiatric phenotypes can improve the predictive performance of models trained on clinical data (EHR + survey) alone.

The incorporation of PRSs into data-driven prediction models could be justified if PRSs sufficiently improved predictive performance and were paired with evidence-based interventions. Although integrating PRSs into clinical workflows presents implementation challenges, there is increasing momentum toward the broad implementation of genomic information in health care practice [4]. As the cost of genome sequencing continues to decrease, genomic data are expected to ultimately become a standard component of patient health care records. The goal of this paper was to provide a first look at whether such information might in fact provide predictive enhancements that could justify its use.

## Methods

### Sample

Eligible patients for this study were those who participated in our previous study [3] of adult patients visiting the ED between February 4, 2015, and March 13, 2017; had their blood samples genotyped through their participation in the Mass General Brigham (MGB) Biobank [5] (88% self-reported White); and had nonmissing information on suicide attempt(s) within 6 months following their ED discharge. In total, 333 patients with genetically identified European ancestry met the eligibility criteria and demonstrated a suicide attempt prevalence of 8.4% (n=28) at the 6-month follow-up (n=178, 53.5% self-reported male and n=324, 97.3% self-reported White). Although our previous study [3] also examined suicide attempts at 1 month after ED discharge, the event rate within this window was too low to permit stable estimates. The study sample differed significantly from the original cohort [3] by age (*P*<.001), self-reported race (*P*<.001) and ethnicity (*P*=.06), insurance type (*P*=.001), and patterns of health care utilization (*P*<.001; see Multimedia Appendix 1 [3]). Details on recruitment, informed consent process, and data collection can be found in Boutin et al [5] (for the MGB Biobank study) and Nock et al [3] (for the suicide prediction study).

### Outcome

The primary outcome was any suicide attempt within 6 months of the ED visit based on either follow-up surveys or a review of linked EHRs [3]. For the latter, we used the *International Classification of Diseases, Ninth Revision* (*ICD-9*) and *International Classification of Diseases, Tenth Revision* (*ICD-10*) to identify qualifying diagnostic codes for suicide attempts that we previously validated [6,7].

### Predictors

We extracted the predicted probabilities from the best-performing ensemble model from our previous work [3] for 6-month suicide attempts. This model incorporated patient-reported surveys, a previously developed EHR-based suicide risk score, and sociodemographic characteristics (eg, age, sex, income, education, race and ethnicity, and employment status). In addition, we generated PRSs for depression, bipolar disorder, schizophrenia, suicide attempt, and externalizing traits derived from the largest available European ancestry genome-wide association study of these phenotypes using a Bayesian polygenic risk scoring method called “PRS-CS” (see Multimedia Appendices 2 and 3) [8]. We subsequently residualized individual disorder PRSs for biological sex, age, genomic chip, and the top 20 principal components for population stratification to adjust for potential confounding.

### Statistical Analysis

We first established the baseline model by fitting our previously validated suicide risk score and calculated patient risk stratification accuracy (measured using the area under the receiver operating characteristic curve [AUC], area under the precision-recall curve [AUPRC], and positive predictive value [PPV]). We then added each PRS to the baseline model to evaluate whether adding individual disorder PRSs would improve the AUC, AUPRC, or PPV. Lastly, we incorporated all 5 PRSs to examine whether incorporating multiple neuropsychiatric PRSs would increase the predetermined metrics more than adding individual disorder PRSs to the baseline model alone.

In addition to fitting logistic regression models, we used the SuperLearner stacked generalization approach that combines predictions across a range of algorithms, including those that can capture nonlinear relationships (see Multimedia Appendix 4) [9]. We used 10-fold stratified cross-validation in a 70% training sample (n_train_=235) to develop the models and evaluated the models in a 30% holdout sample (n_holdout_=98). There were no significant differences in sample characteristics and feature distributions between the train and holdout samples (all *P*>.05; see Multimedia Appendix 5). All statistical analyses were conducted using R software (version 4.1.2; R Foundation for Statistical Computing).

### Ethical Considerations

The study procedures were approved by the Institutional Review Boards of Harvard University and MGB (protocol code 2010P000246, approved on February 18, 2010). Additionally, the MGB Biobank study was conducted in accordance with the Declaration of Helsinki and approved by the MGB Institutional Review Board (protocol code 2009P002312, approved on April 29, 2010), with no compensation provided to participants. This study involves secondary analyses using de-identified data from the original studies, which is covered under the initial consent and IRB approval, without requiring additional consent.

## Results

### Model Discrimination

**Figure 1:**
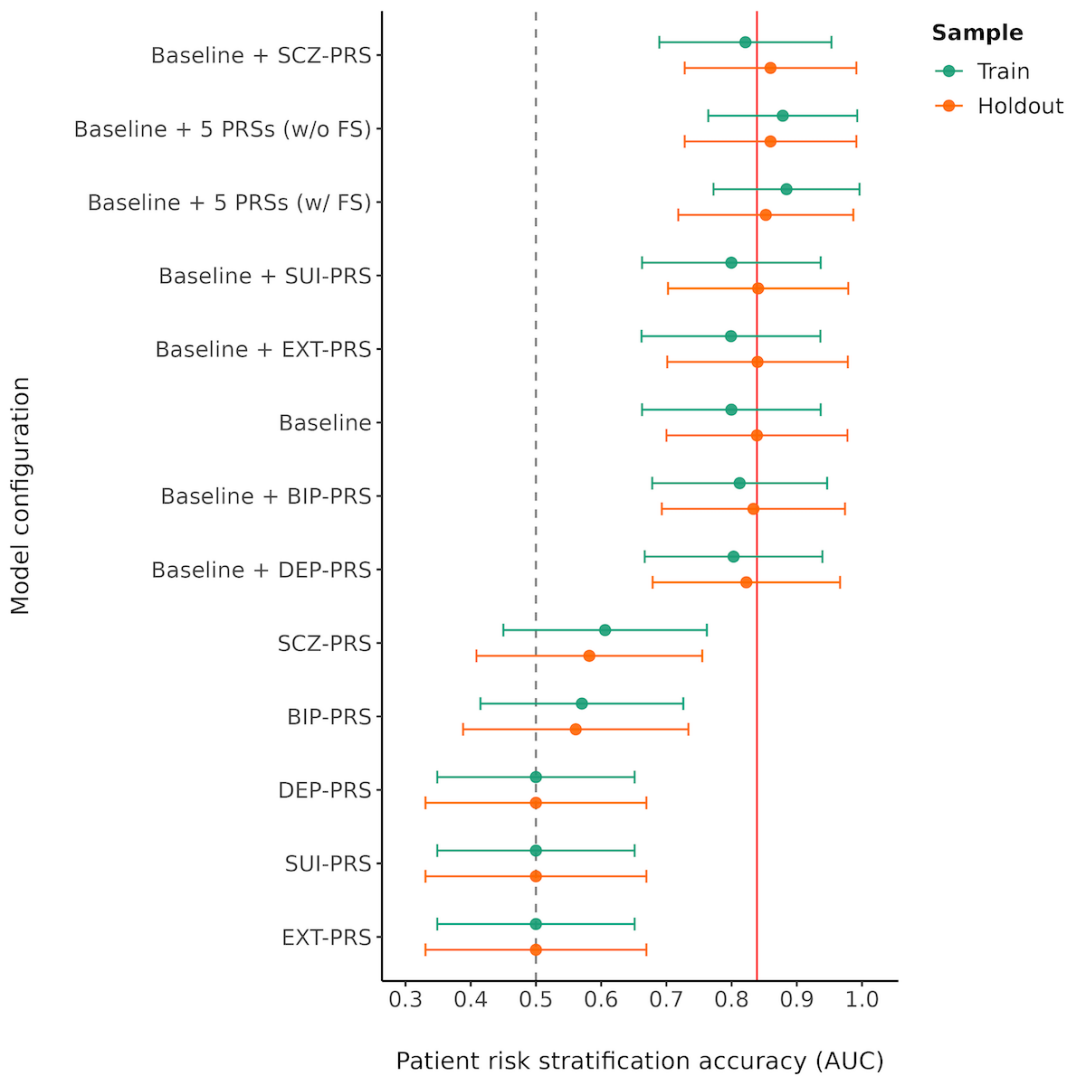
Patient risk stratification accuracy from SuperLearner models estimated using the train (in green) and holdout (in orange) samples. The y-axis is sorted based on the AUC point estimates in the holdout sample. The red line represents the reference AUC point estimate from the baseline model in the holdout sample and is depicted to facilitate visual comparison of AUC estimates across different model configurations. Baseline: baseline clinical risk score for suicide attempt; BIP: bipolar disorder; DEP: depression; EXT: externalizing traits; PRS: polygenic risk score; SCZ: schizophrenia; SUI: suicide attempt; w: with; w/o: without.

The baseline model for 6-month suicide attempts had an AUC of 0.84 (95% CI 0.70-0.98; see Figure 1 and Multimedia Appendix 6)**.** Models that included individual disorder PRSs alone had modest or poor AUC, with the schizophrenia PRS having the highest AUC (0.58, 95% CI 0.41-0.76), followed by the bipolar disorder PRS (0.56, 95% CI 0.39-0.73). When individual disorder PRSs were added to the baseline model, the logistic regression and the ensemble models that included the schizophrenia PRS and clinical risk score had the highest AUC (0.86, 95% CI 0.73-0.99), followed by ensemble models each including the suicide PRS and externalizing disorder PRS, but these provided only a modest numerical increase in AUC compared to the baseline model alone (see Figure 1). In general, there was no improvement in AUC when adding the PRS for depression or bipolar disorder to the clinical risk score. However, we observed a numerically higher AUC when the depression PRS was incorporated using an ensemble approach than using logistic regression. The ensemble model that included the clinical risk score and all 5 PRSs had the same AUC (0.86, 95% CI 0.72-0.99) as the ensemble model including the schizophrenia PRS and clinical risk score and had nearly the same AUC as the logistic regression including the same set of features.

### Model Performance

**Figure 2:**
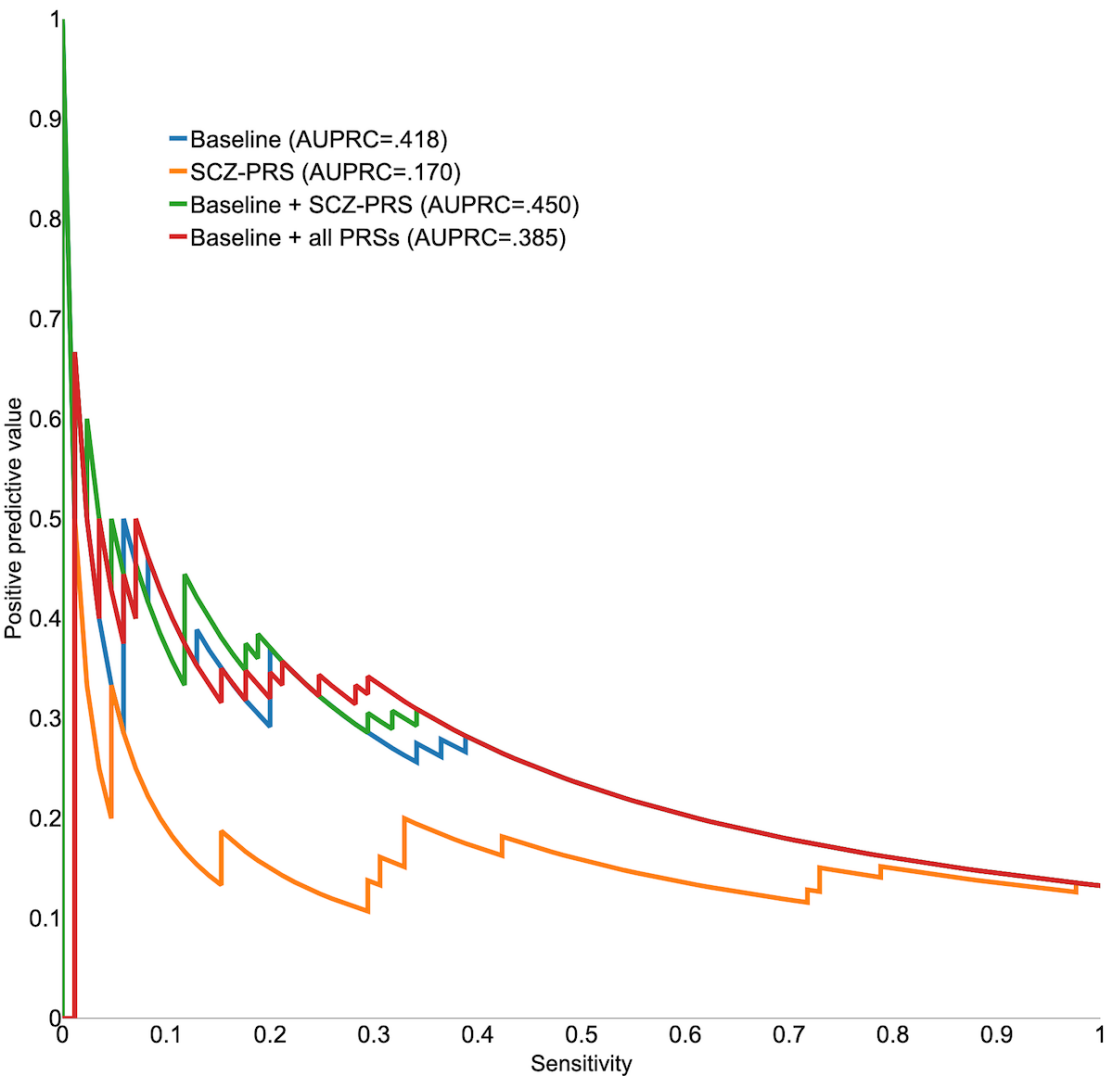
A precision-recall curve for predicting suicide attempt within 6 months after an ED discharge. AUPRC: area under the precision-recall curve; ED: emergency department; PRS: polygenic risk score; SCZ: schizophrenia.

We examined precision-recall curves to see how PPV varies across levels of sensitivity with the goal of explaining the best-performing model, which included the clinical risk score and schizophrenia PRS (see Figure 2). All models that included the clinical risk score were comparable in identifying 40% to 50% of suicide attempt cases within 6 months after ED discharge, indicating a higher sensitivity than the models only including individual disorder PRSs (see Multimedia Appendix 7). Specifically, shown in Figure 2, the baseline model had a higher PPV (26%-50%) than the other models when the sensitivity was in the 0.05 to 0.35 range. The models including the clinical risk score with or without PRSs had the same PPV (13%-26%) when the sensitivity was in the 0.4 to 1.0 range, and the model with the schizophrenia PRS alone had a lower PPV (12%-18%). AUPRC was 0.42 for the baseline model but reached 0.45 when the schizophrenia PRS was added, which is consistent with the observed improvement in AUC with the same model configuration.

## Discussion

### Principal Findings

We found modest evidence suggesting that the integration of the PRS for schizophrenia (but the PRSs for not the other related phenotypes) might enhance the prediction of short-term risk for suicide attempt in patients discharged from the ED; both the AUC and AUPRC were numerically, although not significantly, higher when the schizophrenia PRS was added to the baseline clinical model. The improved predictive performance is likely explained by the higher heritability and statistical power of the schizophrenia PRS compared to the other PRSs examined in this study (see Multimedia Appendix 8). However, while heritability provides a compelling explanation, it does not fully account for the schizophrenia findings, as the predictive power of PRSs is also influenced by factors such as genetic architecture and heterogeneity in phenotype ascertainment. Furthermore, given the high dimensionality of the phenotypic features in the suicide prediction model, the addition of 1 or more PRSs is expected to have only a modest effect on prediction accuracy.

### Limitations

Nevertheless, the nonsignificant improvement in performance we observed should be interpreted in light of our limited study sample size and statistical power of neuropsychiatric PRSs. Of the PRSs we examined, only the schizophrenia PRS was well powered (88%) to detect an association with suicide attempt in the holdout sample.

### Future Work

Future studies utilizing larger biobank samples will enable a more robust and well-powered evaluation of the potential utility of PRSs in enhancing patient risk stratification in high-risk clinical settings. For instance, larger samples could facilitate the training of separate, context-specific baseline models using EHR and survey data from patients with schizophrenia or bipolar disorder, followed by the integration of the respective PRSs into each model. Such an approach would provide a more nuanced understanding of the clinical relevance of PRSs and their potential role in improving risk stratification and patient outcomes.

### Conclusions

In conclusion, we did not observe a substantial benefit of adding psychiatric PRSs to EHR and survey-based prediction models of suicide attempt in an ED setting. Given the importance of optimizing risk stratification to inform suicide prevention, further studies in large, diverse samples are warranted to clarify the value of incorporating genomic risk factors.

## Appendix

Multimedia Appendix 1 Comparison of demographic and clinical characteristics of the study population relative to the original population in Nock et al (2022).

Multimedia Appendix 2 Supplemental methods.

Multimedia Appendix 3 A list of genome-wide association study summary statistics used for polygenic risk score calculation.

Multimedia Appendix 4 Ensemble weights and cross-validated risk sorted in descending order of ensemble weights and risk.

Multimedia Appendix 5 Demographic and clinical characteristics of the study population, stratified by train-holdout split.

Multimedia Appendix 6 Patient risk stratification accuracy from SuperLearner models in the holdout sample.

Multimedia Appendix 7 Sensitivity and positive predictive value of the ensemble models predicting a suicide attempt within 6 months of emergency department discharge in the holdout sample based on the baseline model and the best-performing model.

Multimedia Appendix 8 Power curves for univariate associations of 5 polygenic risk scores with suicide attempt in the holdout sample.

## Abbreviations

AUC: area under the receiver operator curve
AUPRC: area under the precision-recall curve
ED: emergency department
EHR: electronic health record
*ICD-9*: *International Classification of Diseases, Ninth Revision*
*ICD-10*: *International Classification of Diseases, Tenth Revision*
MGB: Mass General Brigham
PPV: positive predictive value
PRS: polygenic risk score
