# Supplementary material for: Enhancing Suicide Risk Prediction With Polygenic Scores in Psychiatric Emergency Settings: Prospective Study
Source: JMIR Bioinform Biotechnol. 2024 Oct 23;5:e58357. doi: 10.2196/58357 (PMC11541145; doi:10.2196/58357)
Supplement: Multimedia Appendix 1 [file bioinform_v5i1e58357_app1.docx]

|  | **6-month follow-up in Nock et al [1], 2022^a^** (N=1,149) | **Study sample** (N=333, 29.0%) | ***P*** |
| --- | --- | --- | --- |
| **Prevalence of suicide attempt at the 6-month follow up (%)** | | | |
|  | 77 (6.7) | 28 (8.4) | .18 |
| **Self-reported gender (%)** | | | |
| Male | 581 (50.6) | 178 (53.5) | .24 |
| Female | 568 (49.4) | 155 (46.5) |  |
| **Self-reported race (%)** | | | |
| Asian | 43 (3.7) | 0 (0.0) | <.001 |
| Black | 113 (9.8) | 0 (0.0) |  |
| Other | 129 (11.2) | 9 (2.7) |  |
| White | 864 (75.2) | 324 (97.3) |  |
| **Self-reported ethnicity (%)** | | | |
| Hispanic | 12 (1.0) | 0 (0.0) | .06 |
| Non-Hispanic | 1137 (99.0) | 333 (100.0) |  |
| **Marital status (%)** | | | |
| Divorced | 100 (8.7) | 33 (9.9) | .26 |
| Married | 152 (13.2) | 53 (15.9) |  |
| Other/Unknown | 22 (1.9) | 4 (1.2) |  |
| Partner | 13 (1.1) | 2 (0.6) |  |
| Separated | 22 (1.9) | 4 (1.2) |  |
| Single | 823 (71.6) | 231 (69.4) |  |
| Widowed | 17 (1.5) | 6 (1.8) |  |
| **Insurance type (%)** | | | |
| Public | 835 (72.7) | 266 (79.9) | .001 |
| Private | 314 (27.3) | 67 (20.1) |  |
| **Continuous variables [mean (SD)]** | | | |
| Age at study baseline | 34.35 (13.6) | 33.37 (13.48) | <.001 |
| Baseline suicide risk (0 to 1) | 0.21 (0.21) | 0.20 (0.19) | <.001 |
| **Healthcare utilization [median (IQR)]** | | | |
| Visit count | 99.00 [27.00, 270.00] | 76.50 [21.00, 227.25] | <.001 |
| ICD code count | 254.00 [63.00, 669.00] | 180.00 [49.00, 542.50] | <.001 |
| Note count | 540.00 [130.00, 1,670.00] | 412.50 [95.00, 1,350.75] | <.001 |

^a^ Of the 1,220 participants in the 6-month follow-up survey, 69 did not have matching electronic health records (EHR), and an additional 2 had missing demographic information in their EHR. Consequently, this resulted in a total of 1,149 participants being included in the demographic comparison between the Nock et al., 2022, cohort and the current study sample.

1. *Nock MK, Millner AJ, Ross EL, Kennedy CJ, Al-Suwaidi M, Barak-Corren Y, et al. Prediction of suicide attempts using clinician assessment, patient self-report, and electronic health records. JAMA Netw Open. 2022;5: e214437 PMID: 35084483, PMCID: PMC8796020.*
