# Supplementary material for: Enhancing Suicide Risk Prediction With Polygenic Scores in Psychiatric Emergency Settings: Prospective Study
Source: JMIR Bioinform Biotechnol. 2024 Oct 23;5:e58357. doi: 10.2196/58357 (PMC11541145; doi:10.2196/58357)
Supplement: Multimedia Appendix 2 [file bioinform_v5i1e58357_app2.docx]

## **Supplemental Methods**

**Genotyping QC pipeline**

The first subset of MGB Biobank samples (N_Initial, MEGA_ = 36,424; hereafter, MEGA samples) were genotyped on Multi-Ethnic Global Array (MEGA) kits from Illumina (Illumina Inc., San Diego, USA) and released in eight batches. We performed batch-specific genotype data QC to remove single nucleotide polymorphisms (SNPs) with genotype missing rate >0.05, samples with genotype missing rate >0.02, and SNPs with differential missing rate >0.01 between any two batches, after which different batches were merged for subsequent QC steps.

As MGB Biobank included individuals from diverse populations, we inferred the genetic ancestry of biobank participants using 1000 Genomes samples (1KG) as the population reference panel (1000 Genomes Project Consortium et al., 2015). Specifically, we computed principal components (PCs) for biobank samples and 1KG samples combined and trained a random forest classifier to assign a “super population” label for biobank samples with a prediction probability ≥0.9 using the first 6 PCs of the 1KG samples as the training data. This resulted in 25,677 individuals whose ancestry was classified as European (EUR), 1,607 as African (AFR), 1,840 as Admixed American (AMR), 504 as East Asian (EAS), and 297 as South Asian (SAS) ancestry.

The second subset of MGB Biobank samples (N_Initial, GSA_ = 47,321; hereafter, GSA samples) were genotyped on Global Screening Array (GSA) kits from Illumina (Illumina Inc., San Diego, USA) and released in three batches. We performed batch-specific genotype data QC and computed PCs using the same criteria and procedures as was done in the MEGA samples. This resulted in 33,614 individuals whose ancestry was classified as EUR, 2,036 as AFR, 2,599 as AMR, 908 as EAS, and 480 as SAS ancestry.

The following procedures were applied to both MEGA and GSA samples. Within each ancestry, we excluded samples with mismatched reported and genetic sex, outliers of the absolute value of heterozygosity (>5 standard deviations from the mean), and one sample from each pair of related individuals (identity-by-descent (IBD) >0.2); SNPs that showed significant batch associations at P < 1 × 10−4, had a missing rate > 0.02 or Hardy–Weinberg equilibrium (HWE) test P < 1 × 10−10 were also discarded. Next, we used the Michigan Imputation Server (Minimac4) to impute genotype dosages for biobank samples, with the Haplotype Reference Consortium (HRC) as the reference panel for EUR ancestry. Lastly, we removed markers with imputation quality INFO score <0.8, minor allele frequency (MAF) <0.01, a significant deviation from HWE with P < 1 × 10−10, and missing rate >0.02. The dataset uses genome build 37 (hg19). Further information about genotyping, QC, imputation, and population assignment procedures for the MGB Biobank is available on the GitHub repositories for the respective samples (MEGA samples: <https://github.com/Annefeng/PBK-QC-pipeline>, GSA samples: <https://github.com/getian107/MGBB-QC>).

**Approach to deduplicating genomic samples**

In our study sample of 333 participants, 171 participants (118 in the train sample and the remaining 53 in the holdout sample) had DNA genotyped using both MEGA and GSA kits, resulting in duplicate genotype data. With MEGA providing a denser array with approximately 1.7 million variants as opposed to the GSA’s coverage of around 660,000 variants, we prioritized the MEGA samples for their broader variant coverage.[1]

**Construction of Bayesian polygenic risk scores (PRS)**

We generated PRS for the European ancestry participants of the MGB Biobank using PRS-CS-Auto [2], a Bayesian polygenic prediction method, based on their genotype data and publicly available summary statistics from the largest, European ancestry genome-wide association studies (GWAS) of five neuropsychiatric traits shown in eTable 2. PRS-CS-Auto places a continuous shrinkage (CS) prior on SNP effect sizes and infers posterior SNP weights using GWAS summary statistics and an external linkage disequilibrium (LD) reference panel [3]. Allowing multivariate modeling of local LD patterns, PRS-CS-Auto is robust to diverse underlying genetic architectures and can increase the accuracy of PRS over conventional approaches [2]. We generated PRS for each individual by summing all risk-associated variants weighted by their posterior effect size estimates inferred by PRS-CS-Auto using PLINK, version 1.9 [4]. Each PRS was subsequently adjusted for biological sex, age, genomic chip, and the top 20 principal components for genetic ancestry to adjust for potential confounding, and standardized to have a mean of 0 and a standard deviation of 1. For each PRS, the standardized residuals were binarized based on the top 10% value of the PRS distribution from the training sample. Participants with a PRS greater than or equal to this threshold (“high genetic risk”) were given a value of 1, while those with a PRS below the threshold were given a value of 0.

**eReferences**

1. Martin AR, Atkinson EG, Chapman SB, Stevenson A, Stroud RE, Abebe T, et al. Low-coverage sequencing cost-effectively detects known and novel variation in underrepresented populations. Am J Hum Genet. 2021;108: 656–668. doi:10.1016/j.ajhg.2021.03.012

2. Ge T, Chen C-Y, Ni Y, Feng Y-CA, Smoller JW. Polygenic prediction via Bayesian regression and continuous shrinkage priors. Nat Commun. 2019;10: 1776. doi:10.1038/s41467-019-09718-5

3. 1000 Genomes Project Consortium, Auton A, Brooks LD, Durbin RM, Garrison EP, Kang HM, et al. A global reference for human genetic variation. Nature. 2015;526: 68–74. doi:10.1038/nature15393

4. Purcell S, Neale B, Todd-Brown K, Thomas L, Ferreira MAR, Bender D, et al. PLINK: a tool set for whole-genome association and population-based linkage analyses. Am J Hum Genet. 2007;81: 559–575. doi:10.1086/519795
