# Supplementary material for: Enhancing Suicide Risk Prediction With Polygenic Scores in Psychiatric Emergency Settings: Prospective Study
Source: JMIR Bioinform Biotechnol. 2024 Oct 23;5:e58357. doi: 10.2196/58357 (PMC11541145; doi:10.2196/58357)
Supplement: Multimedia Appendix 3 [file bioinform_v5i1e58357_app3.docx]

| **Phenotype** | **n_case_** | **n_control_** | **N_total_** | **Author (Year)** | **PMID** |
| --- | --- | --- | --- | --- | --- |
| Bipolar disorder | 41,917 | 371,549 | 413,466 | Mullins (2021) [1] | 34002096 |
| Depression | 170,756 | 329,443 | 500,199 | Howard (2019) [2] | 30718901 |
| Schizophrenia | 53,386 | 77,258 | 130,644 | Trubetskoy (2022) [3] | 35396580 |
| Suicide attempt | 29,782 | 519,961 | 549,743 | Mullins (2022) [4] | 34861974 |
| Externalizing traits | 1,492,085 (with 23andMe) | | | Karlsson-Linner (2021) [5] | 34446935 |
|  | 1,045,957 (without 23andMe) | | | Williams (2023) [6] | 36993611 |

**Note:** We used the summary statistics from Williams 2023 et al. (i.e., the version *excluding* 23andMe data) to calculate PRS for externalizing traits.

**eReferences:**

1. Mullins N, Forstner AJ, O'Connell KS, et al. Genome-wide association study of more than 40,000 bipolar disorder cases provides new insights into the underlying biology. *Nat Genet*. 2021;53(6):817-829. doi:10.1038/s41588-021-00857-4.
2. Howard DM, Adams MJ, Clarke TK, et al. Genome-wide meta-analysis of depression identifies 102 independent variants and highlights the importance of the prefrontal brain regions. *Nat Neurosci*. 2019;22(3):343-352. doi:10.1038/s41593-018-0326-7
3. Trubetskoy V, Pardiñas AF, Qi T, et al. Mapping genomic loci implicates genes and synaptic biology in schizophrenia. *Nature*. 2022;604(7906):502-508. doi:10.1038/s41586-022-04434-5
4. Mullins N, Kang J, Campos AI, et al. Dissecting the Shared Genetic Architecture of Suicide Attempt, Psychiatric Disorders, and Known Risk Factors. *Biol Psychiatry*. 2022;91(3):313-327. doi:10.1016/j.biopsych.2021.05.029
5. Karlsson Linnér R, Mallard TT, Barr PB, et al. Multivariate analysis of 1.5 million people identifies genetic associations with traits related to self-regulation and addiction. *Nat Neurosci*. 2021;24(10):1367-1376. doi:10.1038/s41593-021-00908-3
6. Williams CM, Poore H, Tanksley PT, et al. Guidelines for Evaluating the Comparability of Down-Sampled GWAS Summary Statistics. Preprint. *bioRxiv*. 2023;2023.03.21.533641. Published 2023 Mar 24. doi:10.1101/2023.03.21.533641
