# Supplementary material for: Enhancing Suicide Risk Prediction With Polygenic Scores in Psychiatric Emergency Settings: Prospective Study
Source: JMIR Bioinform Biotechnol. 2024 Oct 23;5:e58357. doi: 10.2196/58357 (PMC11541145; doi:10.2196/58357)
Supplement: Multimedia Appendix 4 [file bioinform_v5i1e58357_app4.docx]

| **N of features** | **Feature(s)** | **Learner** | **Weights** | **CV risk** |
| --- | --- | --- | --- | --- |
| 1 | Baseline | Generalized additive model | 0.616 | 0.057 |
|  | Baseline | Earth (tuned) | 0.198 | 0.058 |
|  | Baseline | Outcome mean | 0.186 | 0.060 |
|  | Baseline | Random forest | 0.000 | 0.095 |
|  | Baseline | Logistic regression | 0.000 | 0.058 |
|  | Baseline | Generalized additive model^b^ | 0.000 | 0.058 |
|  | DEP-PRS | Earth (tuned) | 1.000 | 0.060 |
|  | DEP-PRS | Outcome mean | 0.000 | 0.060 |
|  | DEP-PRS | Logistic regression | 0.000 | 0.061 |
|  | DEP-PRS | Generalized additive model | 0.000 | 0.061 |
|  | DEP-PRS | Generalized additive model^b^ | 0.000 | 0.061 |
|  | DEP-PRS | Random forest | 0.000 | 0.061 |
|  | BIP-PRS | Earth (tuned) | 0.526 | 0.060 |
|  | BIP-PRS | Random forest | 0.474 | 0.060 |
|  | BIP-PRS | Outcome mean | 0.000 | 0.060 |
|  | BIP-PRS | Logistic regression | 0.000 | 0.060 |
|  | BIP-PRS | Generalized additive model | 0.000 | 0.060 |
|  | BIP-PRS | Generalized additive model^b^ | 0.000 | 0.060 |
|  | SCZ-PRS | Generalized additive model | 0.406 | 0.059 |
|  | SCZ-PRS | Random forest | 0.339 | 0.060 |
|  | SCZ-PRS | Outcome mean | 0.256 | 0.060 |
|  | SCZ-PRS | Earth (tuned) | 0.000 | 0.061 |
|  | SCZ-PRS | Logistic regression | 0.000 | 0.059 |
|  | SCZ-PRS | Generalized additive model^b^ | 0.000 | 0.059 |
|  | SUI-PRS | Earth (tuned) | 1.000 | 0.060 |
|  | SUI-PRS | Outcome mean | 0.000 | 0.060 |
|  | SUI-PRS | Logistic regression | 0.000 | 0.060 |
|  | SUI-PRS | Generalized additive model | 0.000 | 0.060 |
|  | SUI-PRS | Generalized additive model^b^ | 0.000 | 0.060 |
|  | SUI-PRS | Random forest | 0.000 | 0.060 |
|  | EXT-PRS | Earth (tuned) | 1.000 | 0.060 |
|  | EXT-PRS | Logistic regression | 0.000 | 0.061 |
|  | EXT-PRS | Generalized additive model | 0.000 | 0.061 |
|  | EXT-PRS | Generalized additive model^b^ | 0.000 | 0.061 |
|  | EXT-PRS | Random forest | 0.000 | 0.061 |
|  | EXT-PRS | Outcome mean | 0.000 | 0.060 |

| 2 | Baseline + DEP-PRS | Generalized additive model | 0.653 | 0.058 |
| --- | --- | --- | --- | --- |
|  | Baseline + DEP-PRS | Outcome mean | 0.255 | 0.060 |
|  | Baseline + DEP-PRS | Logistic regression | 0.092 | 0.058 |
|  | Baseline + DEP-PRS | Logistic regression^a^ | 0.000 | 0.061 |
|  | Baseline + DEP-PRS | Lasso regression | 0.000 | 0.059 |
|  | Baseline + DEP-PRS | Ridge regression | 0.000 | 0.058 |
|  | Baseline + DEP-PRS | Generalized additive model^b^ | 0.000 | 0.058 |
|  | Baseline + DEP-PRS | Earth (tuned) | 0.000 | 0.059 |
|  | Baseline + DEP-PRS | Random forest | 0.000 | 0.061 |
|  | Baseline + BIP-PRS | Generalized additive model | 0.766 | 0.057 |
|  | Baseline + BIP-PRS | Outcome mean | 0.234 | 0.060 |
|  | Baseline + BIP-PRS | Logistic regression | 0.000 | 0.058 |
|  | Baseline + BIP-PRS | Logistic regression^a^ | 0.000 | 0.059 |
|  | Baseline + BIP-PRS | Lasso regression | 0.000 | 0.059 |
|  | Baseline + BIP-PRS | Ridge regression | 0.000 | 0.058 |
|  | Baseline + BIP-PRS | Generalized additive model^b^ | 0.000 | 0.058 |
|  | Baseline + BIP-PRS | Earth (tuned) | 0.000 | 0.059 |
|  | Baseline + BIP-PRS | Random forest | 0.000 | 0.061 |
|  | Baseline + SCZ-PRS | Generalized additive model | 0.676 | 0.058 |
|  | Baseline + SCZ-PRS | Outcome mean | 0.292 | 0.060 |
|  | Baseline + SCZ-PRS | Logistic regression^a^ | 0.032 | 0.061 |
|  | Baseline + SCZ-PRS | Logistic regression | 0.000 | 0.059 |
|  | Baseline + SCZ-PRS | Lasso regression | 0.000 | 0.058 |
|  | Baseline + SCZ-PRS | Ridge regression | 0.000 | 0.058 |
|  | Baseline + SCZ-PRS | Generalized additive model^b^ | 0.000 | 0.058 |
|  | Baseline + SCZ-PRS | Earth (tuned) | 0.000 | 0.059 |
|  | Baseline + SCZ-PRS | Random forest | 0.000 | 0.060 |
|  | Baseline + SUI-PRS | Lasso regression | 0.805 | 0.058 |
|  | Baseline + SUI-PRS | Generalized additive model | 0.128 | 0.058 |
|  | Baseline + SUI-PRS | Outcome mean | 0.067 | 0.060 |
|  | Baseline + SUI-PRS | Logistic regression | 0.000 | 0.059 |
|  | Baseline + SUI-PRS | Logistic regression^a^ | 0.000 | 0.060 |
|  | Baseline + SUI-PRS | Ridge regression | 0.000 | 0.058 |
|  | Baseline + SUI-PRS | Generalized additive model^b^ | 0.000 | 0.059 |
|  | Baseline + SUI-PRS | Earth (tuned) | 0.000 | 0.059 |
|  | Baseline + SUI-PRS | Random forest | 0.000 | 0.061 |
|  | Baseline + EXT-PRS | Lasso regression | 0.513 | 0.058 |
|  | Baseline + EXT-PRS | Generalized additive model | 0.305 | 0.058 |
|  | Baseline + EXT-PRS | Outcome mean | 0.181 | 0.060 |
|  | Baseline + EXT-PRS | Logistic regression | 0.000 | 0.059 |
|  | Baseline + EXT-PRS | Logistic regression^a^ | 0.000 | 0.061 |
|  | Baseline + EXT-PRS | Ridge regression | 0.000 | 0.058 |
|  | Baseline + EXT-PRS | Generalized additive model^b^ | 0.000 | 0.059 |
|  | Baseline + EXT-PRS | Earth (tuned) | 0.000 | 0.059 |
|  | Baseline + EXT-PRS | Random forest | 0.000 | 0.061 |
| 6 | Baseline + 5 PRS (w/o FS) | Outcome mean | 0.399 | 0.060 |
|  | Baseline + 5 PRS (w/o FS) | Earth (tuned) | 0.273 | 0.059 |
|  | Baseline + 5 PRS (w/o FS) | Generalized additive model | 0.235 | 0.060 |
|  | Baseline + 5 PRS (w/o FS) | Logistic regression^a^ | 0.093 | 0.091 |
|  | Baseline + 5 PRS (w/o FS) | Logistic regression | 0.000 | 0.062 |
|  | Baseline + 5 PRS (w/o FS) | Lasso regression | 0.000 | 0.060 |
|  | Baseline + 5 PRS (w/o FS) | Ridge regression | 0.000 | 0.059 |
|  | Baseline + 5 PRS (w/o FS) | Generalized additive model^b^ | 0.000 | 0.060 |
|  | Baseline + 5 PRS (w/o FS) | Random forest | 0.000 | 0.064 |
|  | Baseline + 5 PRS (w/ FS) | Generalized additive model^d^ | 0.515 | 0.058 |
|  | Baseline + 5 PRS (w/ FS) | Outcome mean | 0.302 | 0.060 |
|  | Baseline + 5 PRS (w/ FS) | Logistic regression^a^ | 0.095 | 0.091 |
|  | Baseline + 5 PRS (w/ FS) | Logistic regression^a^,^c^ | 0.088 | 0.060 |
|  | Baseline + 5 PRS (w/ FS) | Logistic regression | 0.000 | 0.062 |
|  | Baseline + 5 PRS (w/ FS) | Logistic regression^c^ | 0.000 | 0.060 |
|  | Baseline + 5 PRS (w/ FS) | Logistic regression^d^ | 0.000 | 0.062 |
|  | Baseline + 5 PRS (w/ FS) | Logistic regression^a^,^d^ | 0.000 | 0.091 |
|  | Baseline + 5 PRS (w/ FS) | Lasso regression | 0.000 | 0.059 |
|  | Baseline + 5 PRS (w/ FS) | Lasso regression^c^ | 0.000 | 0.061 |
|  | Baseline + 5 PRS (w/ FS) | Lasso regression^d^ | 0.000 | 0.059 |
|  | Baseline + 5 PRS (w/ FS) | Ridge regression | 0.000 | 0.059 |
|  | Baseline + 5 PRS (w/ FS) | Ridge regression^c^ | 0.000 | 0.060 |
|  | Baseline + 5 PRS (w/ FS) | Ridge regression^d^ | 0.000 | 0.059 |
|  | Baseline + 5 PRS (w/ FS) | Generalized additive model | 0.000 | 0.060 |
|  | Baseline + 5 PRS (w/ FS) | Generalized additive model^d^ | 0.000 | 0.060 |
|  | Baseline + 5 PRS (w/ FS) | Generalized additive model^b^ | 0.000 | 0.060 |
|  | Baseline + 5 PRS (w/ FS) | Generalized additive model^b^,^c^ | 0.000 | 0.058 |
|  | Baseline + 5 PRS (w/ FS) | Generalized additive model^b^,^d^ | 0.000 | 0.060 |
|  | Baseline + 5 PRS (w/ FS) | Earth (tuned) | 0.000 | 0.059 |
|  | Baseline + 5 PRS (w/ FS) | Earth (tuned)^c^ | 0.000 | 0.059 |
|  | Baseline + 5 PRS (w/ FS) | Earth (tuned)^d^ | 0.000 | 0.059 |
|  | Baseline + 5 PRS (w/ FS) | Random forest | 0.000 | 0.064 |
|  | Baseline + 5 PRS (w/ FS) | Random forest^c^ | 0.000 | 0.064 |
|  | Baseline + 5 PRS (w/ FS) | Random forest^d^ | 0.000 | 0.063 |

**Abbreviations:** Baseline, baseline clinical risk score for suicide attempt; PRS, polygenic risk score; SCZ, schizophrenia; SUI, suicide; EXT, externalizing traits; BIP, bipolar disorder; DEP, depression; FS, feature selection.

**Symbols:**

^a^ Fitted with interaction terms.

^b^ Fitted with Mixed Generalized Additive Model Computation Vehicle with Automatic Smoothness Estimation (MGCV).

^c^ Fitted with correlation significance screening to retrain predictors with p < 0.1.

^d^ Fitted with correlation ranking screening to retain the top three predictors.
