# Supplementary material for: Enhancing Suicide Risk Prediction With Polygenic Scores in Psychiatric Emergency Settings: Prospective Study
Source: JMIR Bioinform Biotechnol. 2024 Oct 23;5:e58357. doi: 10.2196/58357 (PMC11541145; doi:10.2196/58357)
Supplement: Multimedia Appendix 5 [file bioinform_v5i1e58357_app5.docx]

|  | **Overall**  (N=333) | **Train**  (n=235, 70.6%) | **Holdout**  (n=98, 29.4%) | ***P*** |
| --- | --- | --- | --- | --- |
| **Self-reported gender (%)** | | | | |
| Male | 178 (53.5) | 123 (52.3) | 55 (56.1) | .61 |
| Female | 155 (46.5) | 112 (47.7) | 43 (43.9) |  |
| **Self-reported race (%)** | | | | |
| White | 324 (97.3) | 228 (97.0) | 96 (98.0) | .91 |
| Other | 9 (2.7) | 7 (3.0) | 2 (2.0) |  |
| **Marital status (%)** | | | | |
| Divorced | 33 (9.9) | 21 (8.9) | 12 (12.2) | .48 |
| Married | 53 (15.9) | 34 (14.5) | 19 (19.4) |  |
| Single | 231 (69.4) | 169 (71.9) | 62 (63.3) |  |
| Other^a^ | 16 (4.8) | 11 (4.8) | 5 (5.0) |  |
| **Insurance type (%)** | | | | |
| Public | 266 (79.9) | 189 (80.4) | 77 (78.6) | .81 |
| Private | 67 (20.1) | 46 (19.6) | 21 (21.4) |  |
| **Genomic chip** | | | | |
| MEGA^b^ | 116 (34.8) | 82 (34.9) | 34 (34.7) | .99 |
| GSA only | 217 (65.2) | 153 (65.1) | 64 (65.3) |  |
| **Continuous variables [mean (SD)]** | | | | |
| Mean age at study baseline  (range: 23, 90) | 36.8 (13.6) | 35.9 (13.1) | 38.8 (14.6) | .08 |
| Baseline (range: 0.04, 0.91) | 0.25 (0.23) | 0.26 (0.24) | 0.23 (0.19) | .21 |
| DEP-PRS (range: -0.31, 0.20) | 0.02 (0.08) | 0.02 (0.08) | 0.03 (0.08) | .34 |
| SCZ-PRS (range: -1.04, 0.71) | 0.07 (0.33) | 0.07 (0.31) | 0.05 (0.36) | .60 |
| BIP-PRS (range: -0.36, 0.33) | 0.02 (0.11) | 0.02 (0.11) | 0.01 (0.10) | .40 |
| SUI-PRS (range: -0.17, 0.19) | 0.01 (0.05) | 0.01 (0.05) | 0.01 (0.05) | .18 |
| EXT-PRS (range: -0.29, 0.37) | 0.03 (0.11) | 0.03 (0.11) | 0.02 (0.10) | .41 |

^a^ Includes participants who reported other/unknown, living with a partner, separated, and widowed.

^b^ Includes individuals who had MEGA samples only and those who had both MEGA and GSA samples. See Multimedia Appendix 2 for the deduplication strategy.

**Note:** We present raw polygenic risk scores (PRS) before standardization to highlight the variations observed in the original distributions.

**Abbreviations:** Baseline, baseline clinical risk score for suicide attempt; PRS, polygenic risk score; SCZ, schizophrenia; SUI, suicide attempt; EXT, externalizing traits; BIP, bipolar disorder; DEP, depression.
