# Supplementary material for: Enhancing Suicide Risk Prediction With Polygenic Scores in Psychiatric Emergency Settings: Prospective Study
Source: JMIR Bioinform Biotechnol. 2024 Oct 23;5:e58357. doi: 10.2196/58357 (PMC11541145; doi:10.2196/58357)
Supplement: Multimedia Appendix 6 [file bioinform_v5i1e58357_app6.docx]

| **Number of features** | **Model configuration** | **Algorithm** | **AUC  (95% CI)** |
| --- | --- | --- | --- |
| 1 | Baseline | Logistic regression | 0.84 (0.70 - 0.98) |
|  | Baseline | Ensemble | 0.84 (0.70 - 0.98) |
|  | DEP-PRS | Logistic regression | 0.56 (0.39 - 0.73) |
|  | DEP-PRS | Ensemble | 0.50 (0.33 - 0.67) |
|  | BIP-PRS | Logistic regression | 0.56 (0.39 - 0.73) |
|  | BIP-PRS | Ensemble | 0.56 (0.39 - 0.73) |
|  | SCZ-PRS | Logistic regression | 0.58 (0.41 - 0.76) |
|  | SCZ-PRS | Ensemble | 0.58 (0.41 - 0.76) |
|  | SUI-PRS | Logistic regression | 0.51 (0.34 - 0.68) |
|  | SUI-PRS | Ensemble | 0.50 (0.33 - 0.67) |
|  | EXT-PRS | Logistic regression | 0.55 (0.38 - 0.72) |
|  | EXT-PRS | Ensemble | 0.50 (0.33 - 0.67) |
| 2 | Baseline + DEP-PRS | Logistic regression | 0.80 (0.65 - 0.95) |
|  | Baseline + DEP-PRS | Ensemble | 0.83 (0.69 - 0.97) |
|  | Baseline + BIP-PRS | Logistic regression | 0.83 (0.69 - 0.97) |
|  | Baseline + BIP-PRS | Ensemble | 0.83 (0.69 - 0.97) |
|  | Baseline + SCZ-PRS | Logistic regression | 0.86 (0.73 - 0.99) |
|  | Baseline + SCZ-PRS | Ensemble | 0.86 (0.73 - 0.99) |
|  | Baseline + SUI-PRS | Logistic regression | 0.84 (0.70 - 0.98) |
|  | Baseline + SUI-PRS | Ensemble | 0.84 (0.70 - 0.98) |
|  | Baseline + EXT-PRS | Logistic regression | 0.81 (0.66 - 0.95) |
|  | Baseline + EXT-PRS | Ensemble | 0.84 (0.70 - 0.98) |
| 6 | Baseline + 5 PRS | Logistic regression | 0.86 (0.72 - 0.99) |
|  | Baseline + 5 PRS | Ensemble | 0.86 (0.73 - 0.99) |

**Abbreviations:** AUC, area under the receiver operating characteristic curve; CI, confidence interval; Baseline, baseline clinical risk score for suicide attempt; PRS, polygenic risk score; SCZ, schizophrenia; SUI, suicide; EXT, externalizing traits; BIP, bipolar disorder; DEP, depression.
