# Supplementary material for: Enhancing Suicide Risk Prediction With Polygenic Scores in Psychiatric Emergency Settings: Prospective Study
Source: JMIR Bioinform Biotechnol. 2024 Oct 23;5:e58357. doi: 10.2196/58357 (PMC11541145; doi:10.2196/58357)
Supplement: Multimedia Appendix 7 [file bioinform_v5i1e58357_app7.docx]

| **Baseline:** Baseline only | | **Cumulative 6-months suicide attempt** | |
| --- | --- | --- | --- |
| **Decile** | **Case % in Decile** | **SN (SE)** | **PPV (SE)** |
| 0-10% | 38.5 | 38.5 (13.5) | 50.0 (15.8) |
| 10-20% | 15.4 | 53.8 (13.8) | 35.0 (10.7) |
| 20-30% | 23.1 | 76.9 (11.7) | 33.3 (8.6) |
| 30-40% | 0.0 | 76.9 (11.7) | 25.6 (7.0) |
| 40-50% | 23.1 | 100.0 (0.0) | 26.5 (6.3) |
| 50-60% | 0.0 | 100.0 (0.0) | 22.0 (5.4) |
| 60-70% | 0.0 | 100.0 (0.0) | 19.1 (4.8) |
| 70-80% | 0.0 | 100.0 (0.0) | 16.7 (4.2) |
| 80-90% | 0.0 | 100.0 (0.0) | 14.8 (3.8) |
| 90-100% | 0.0 | 100.0 (0.0) | 13.3 (1.2) |
| **PRS only:** SCZ-PRS only | | **Cumulative 6-months suicide attempt** | |
| **Decile** | **Case % in Decile** | **SN (SE)** | **PPV (SE)** |
| 0-10% | 15.4 | 15.4 (10) | 20.0 (12.7) |
| 10-20% | 7.7 | 23.1 (11.7) | 15.0 (8.0) |
| 20-30% | 7.7 | 30.8 (12.8) | 13.3 (6.2) |
| 30-40% | 23.1 | 53.8 (13.8) | 17.9 (6.2) |
| 40-50% | 7.7 | 61.5 (13.5) | 16.3 (5.3) |
| 50-60% | 0.0 | 61.5 (13.5) | 13.6 (4.4) |
| 60-70% | 0.0 | 61.5 (13.5) | 11.8 (3.9) |
| 70-80% | 23.1 | 84.6 (7.9) | 14.1 (3.9) |
| 80-90% | 7.7 | 92.3 (3.9) | 13.6 (3.7) |
| 80-100% | 7.7 | 100.0 (0) | 13.3 (1.2) |
| **Best-performing:** Baseline + SCZ-PRS | | **Cumulative 6-months suicide attempt** | |
| **Decile** | **Case % in Decile** | **SN (SE)** | **PPV (SE)** |
| 0-10% | 30.8 | 30.8 (12.8) | 40.0 (15.5) |
| 10-20% | 30.8 | 61.5 (13.5) | 40.0 (11.0) |
| 20-30% | 15.4 | 76.9 (11.7) | 33.3 (8.6) |
| 30-40% | 15.4 | 92.3 (30.8) | 30.8 (7.4) |
| 40-50% | 7.7 | 100.0 (0.0) | 26.5 (6.3) |
| 50-60% | 0.0 | 100.0 (0.0) | 22.0 (5.4) |
| 60-70% | 0.0 | 100.0 (0.0) | 19.1 (4.8) |
| 70-80% | 0.0 | 100.0 (0.0) | 16.7 (4.2) |
| 80-90% | 0.0 | 100.0 (0.0) | 14.8 (3.8) |
| 90-100% | 0.0 | 100.0 (0.0) | 13.3 (1.2) |

| **All:** Baseline + all PRS | | **Cumulative 6-months suicide attempt** | |
| --- | --- | --- | --- |
| **Decile** | **Case % in Decile** | **SN (SE)** | **PPV (SE)** |
| 0-10% | 30.8 | 30.8 (12.8) | 40.0 (15.5) |
| 10-20% | 23.1 | 53.8 (13.8) | 35.0 (10.7) |
| 20-30% | 23.1 | 76.9 (11.7) | 33.3 (8.6) |
| 30-40% | 23.1 | 100.0 (0) | 33.3 (7.6) |
| 40-50% | 0.0 | 100.0 (0) | 26.5 (6.3) |
| 50-60% | 0.0 | 100.0 (0) | 22 (5.4) |
| 60-70% | 0.0 | 100.0 (0) | 19.1 (4.8) |
| 70-80% | 0.0 | 100.0 (0) | 16.7 (4.2) |
| 80-90% | 0.0 | 100.0 (0) | 14.8 (3.8) |
| 90-100% | 0.0 | 100.0 (0) | 13.3 (1.2) |

**Abbreviations:** Baseline, baseline clinical risk score; PRS, polygenic risk score; SN, sensitivity, SE, standard error; PPV, positive predictive value.
