# Supplementary material for: Enhancing Suicide Risk Prediction With Polygenic Scores in Psychiatric Emergency Settings: Prospective Study
Source: JMIR Bioinform Biotechnol. 2024 Oct 23;5:e58357. doi: 10.2196/58357 (PMC11541145; doi:10.2196/58357)
Supplement: Multimedia Appendix 8 [file bioinform_v5i1e58357_app8.docx]

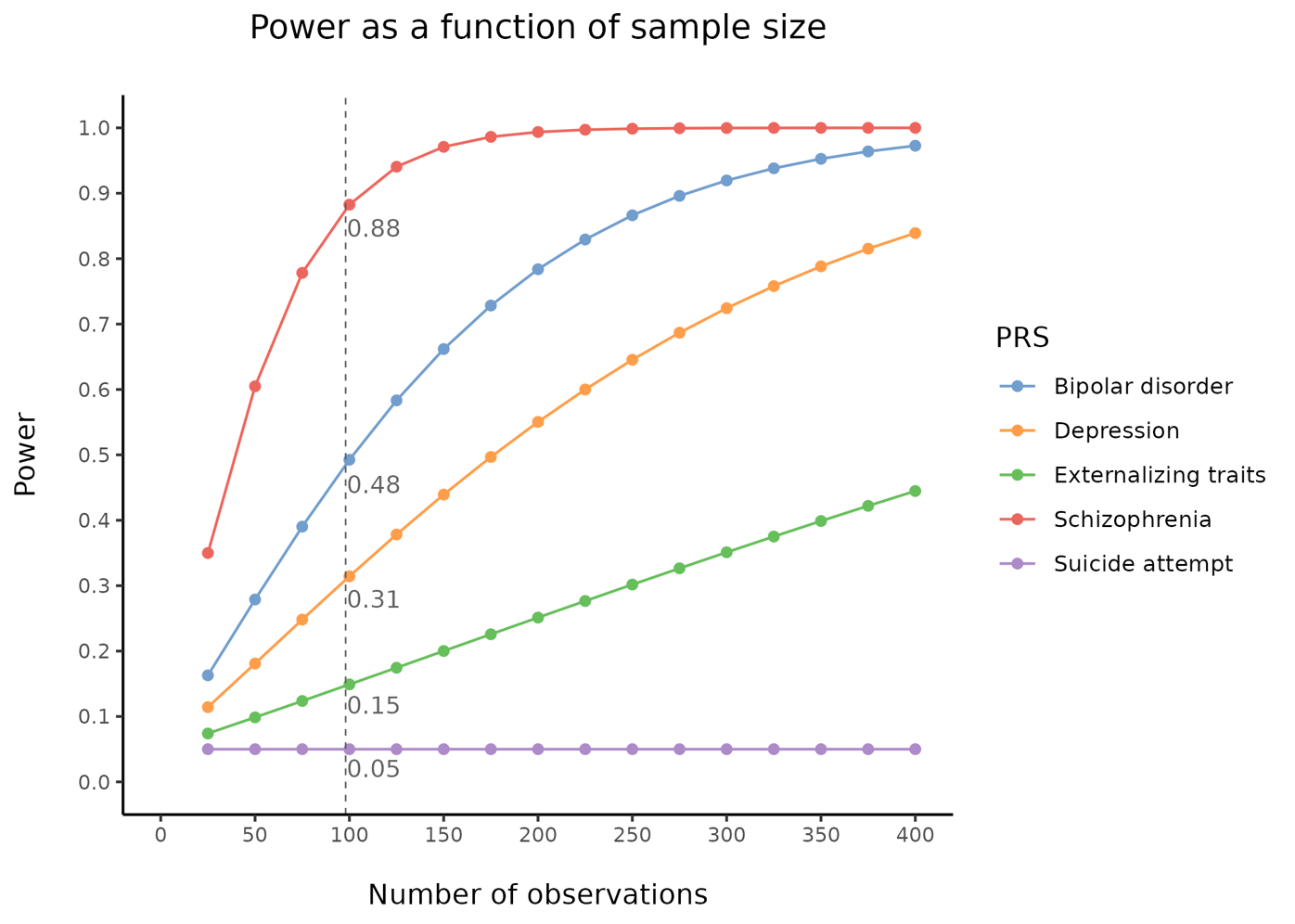


**Note:** The dashed line represents the sample size of the holdout sample (n=98), while annotated values along the curves indicate the observed statistical power for a given PRS in the holdout sample.

**Abbreviations:** PRS, polygenic risk score.
